# A protocol for a multicenter randomized and personalized controlled trial using rTMS in patients with disorders of consciousness

**DOI:** 10.3389/fneur.2023.1216468

**Published:** 2023-07-21

**Authors:** Marie M. Vitello, Martin J. Rosenfelder, Paolo Cardone, Masachika Niimi, Lina Willacker, Aurore Thibaut, Nicolas Lejeune, Steven Laureys, Andreas Bender, Olivia Gosseries

**Affiliations:** ^1^Coma Science Group, GIGA Consciousness, University of Liège, Liège, Belgium; ^2^Centre du Cerveau^2^, University Hospital of Liège, Liège, Belgium; ^3^Department of Neurology, Therapiezentrum Burgau, Burgau, Germany; ^4^Clinical and Biological Psychology, Institute of Psychology and Education, Ulm University, Ulm, Germany; ^5^Department of Rehabilitation Medicine, Nihon University School of Medicine, Tokyo, Japan; ^6^Department of Neurology, Ludwig-Maximilians University Hospital of Munich, University of Munich, Munich, Germany; ^7^William Lennox Neurological Hospital, Ottignies-Louvain-la-Neuve, Belgium; ^8^CERVO Research Center, Laval University, Québec, QC, Canada

**Keywords:** coma, vegetative state, unresponsive wakefulness syndrome, minimally conscious state, dorsolateral prefrontal cortex, angular gyrus, non-invasive brain stimulation, treatment

## Abstract

**Background:**

Improving the functional recovery of patients with DoC remains one of the greatest challenges of the field. Different theories exist about the role of the anterior (prefrontal areas) versus posterior (parietal areas) parts of the brain as hotspots for the recovery of consciousness. Repetitive transcranial magnetic stimulation (rTMS) is a powerful non-invasive brain stimulation technique for the treatment of DoC. However, a direct comparison of the effect of TMS treatment on the front versus the back of the brain has yet to be performed. In this study, we aim to assess the short- and long-term effects of frontal and parietal rTMS on DoC recovery and characterize responders phenotypically.

**Methods/design:**

Ninety patients with subacute and prolonged DoC will be included in a two-part multicenter prospective study. In the first phase (randomized controlled trial, RCT), patients will undergo four rTMS sessions in a crossover design over 10 days, targeting (i) the left dorsolateral prefrontal cortex (DLPFC) and (ii) the left angular gyrus (AG), as well as (iii & iv) their sham alternatives. In the second phase (longitudinal personalized trial), patients will receive personalized stimulations for 20 working days targeting the brain area that showed the best results in the RCT and will be randomly assigned to either active or sham intervention. The effects of rTMS on neurobehavioral and neurophysiological functioning in patients with DoC will be evaluated using clinical biomarkers of responsiveness (i.e., the Coma Recovery Scale-Revised; CRS-R), and electrophysiological biomarkers (e.g., power spectra, functional and effective connectivity, perturbational complexity index before and after intervention). Functional long-term outcomes will be assessed at 3 and 6 months post-intervention. Adverse events will be recorded during the treatment phase.

**Discussion:**

This study seeks to identify which brain region (front or back) is best to stimulate for the treatment of patients with DoC using rTMS, and to characterize the neural correlates of its action regarding recovery of consciousness and functional outcome. In addition, we will define the responders’ profile based on patients’ characteristics and functional impairments; and develop biomarkers of responsiveness using EEG analysis according to the clinical responsiveness to the treatment.

**Clinical Trial Registration:**

https://clinicaltrials.gov/ct2/show/NCT04401319, Clinicaltrials.gov, n° NCT04401319.

## Introduction

### Disorders of consciousness

Severe brain injury may result in disorders of consciousness (DoC) (1). Such neurological conditions range from coma (i.e., no wakefulness and reflex behaviors only), to the unresponsive wakefulness syndrome (UWS/*VS*) (i.e., recovery of wakefulness with reflex behaviors) (1), and the minimally conscious state (MCS) (i.e., reproducible and purposeful behaviors, such as visual pursuit and responses to commands) (2). Moreover, MCS can be subcategorized into MCS- and MCS+ depending on the presence or absence of language processing. MCS- patients can show visual fixation and pursuit, localization of noxious stimuli or emotionally contingent behavior, while MCS+ patients show reproducible command-following, intelligible verbalization or intentional communication (2). Patients are thought to have emerged from MCS when they display functional communication or functional use of two objects in two consecutive assessments (3).

### Therapeutic options in DoC

In the last decade, few studies have investigated treatment options for patients with DoC (4). Recently, some RCTs have been performed, focusing on pharmacological [e.g., amantadine (5), zolpidem (6)] and non-pharmacological interventions to improve patients’ neurobehavioral functioning. Regarding the latter, a recent meta-analysis studying the effect of non-invasive brain stimulation found evidence for left dorsolateral prefrontal cortex (DLPFC) transcranial direct current stimulation efficacy against sham on behavioral measures in MCS patients with low to moderate effect sizes (7).

Among neuromodulation techniques, repetitive transcranial magnetic stimulation (rTMS) is a non-invasive brain stimulation tool that can modulate cortical excitability, enhance neural plasticity, and induce strong neuromodulatory effects that outlast the period of stimulation (8, 9), especially when applied repeatedly. Thus, it is now established that TMS holds an important role in promoting and monitoring functional recovery in severe brain injury (10). In the field of DoC, some studies have investigated rTMS-induced changes on behavior (11) and electrophysiology (12) or both in patients with severe brain damage (12–17). However, these protocols usually differ in several experimental parameters (e.g., stimulation site, stimulation intensity, number of sessions delivered), making it difficult to draw any conclusion on an effective stimulation protocol at this stage.

To date, most studies have investigated the effects of high-frequency (20 Hz) primary motor cortex stimulation to elicit recovery in DoC patients, showing poor to null clinical improvement at the group level (18–20). However, one RCT comparing the effects of 20 Hz stimulation over the motor cortex and the prefrontal cortex to sham stimulation demonstrated improvement in all groups, but of highest magnitude in the motor cortex group (16).

Regarding other target locations, some RCTs recently reported significant clinical changes in patients (i.e., increased behavioral total scores after intervention) when targeting the left prefrontal regions using multiple sessions (i.e., between 10 to 30 sessions) of high-frequency (i.e., 10–20 Hz) rTMS (21–23).

Eventually, two recent open label studies exploring the effect of rTMS over the left parietal cortex found improved behavioral total scores in MCS patients (24) and even in some UWS/*VS* patients (15). Hence, from these studies, it becomes evident that rTMS is feasible in DoC patients, and that some protocols involving specific target parameters may elicit clinical as well as physiological changes (25), especially in the prefrontal and anterior parietal regions (i.e., DLPFC and angular gyrus, AG). However, to our knowledge, although these two stimulation sites seem relevant, there is currently no study comparing the effect of DLPFC versus AG rTMS in DoC patients.

### Consciousness theories to support the role of frontal and parietal rTMS as therapeutic candidates

**Figure 1:**
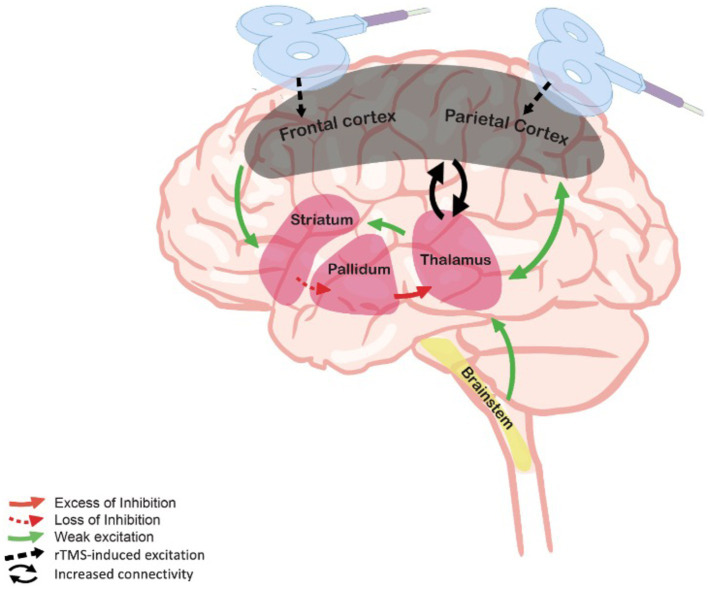
Mesocircuit model and rTMS. The deafferentation of thalamostriatal and corticostriatal outflows following widespread neuronal disruption leads to reduced activity of the striatum, resulting in an inhibition of thalamic activity and decreased thalamo-cortical connectivity and cortical activation. By stimulating the frontal or the parietal cortex, rTMS may hypothetically supply for the missing thalamic excitatory inputs through the reestablishment of cortico-subcortical connectivity. Adapted from Giacino et al. (28).

Despite their indisputable core importance in the dynamic brain processes that are essential in consciousness circuitry, studies trying to isolate the role of frontal versus posterior cortical regions in the emergence of consciousness show contrasting evidence (26). However, it is well established that DoC are caused by widespread dysfunctions preventing the interaction between these areas (27). A common model accounting for post-comatose DoC is the fronto-parietal mesocircuit model (28, 29). This model supports the idea that deafferentation and loss of neurons due to a severe brain injury could induce a reduction of thalamo-cortical and thalamo-striatal functional connectivity from the central thalamus, and consequently, further decreases the activity of the central thalamic and the fronto-parietal networks (30). Figure 1 illustrates the mesocircuit model and the hypothetical changes induced by therapeutic rTMS. Beside this model, the integrated information theory (IIT) postulates that the response of the brain to perturbation needs to be integrated and differentiated – as indexed by the perturbational complexity index (PCI), a proxy for the degree of these components in response to an external perturbation (31). IIT suggests that the posterior part of the brain is the hotspot of consciousness as an experimental hypothesis (26, 27, 32). In contrast, the global neuronal workspace theory suggests that the hotspot of consciousness is located at the front of the brain, and that consciousness arises from ignition (33, 34). In parallel with their theoretical implications, these different models can also guide therapeutic perspectives aimed at restoring consciousness functioning. Thus, approaches acting over these critical structures could hypothetically restore the loops between the central thalamus, the cortex, the striatum and the globus pallidus interna. The loops within and between frontal and parietal cortex can be indexed by EEG-based functional connectivity (35).

In the light of the above theories, we here propose to investigate the effects of rTMS over the frontal and the parietal areas of the brain to promote recovery of consciousness in patients with DoC. More specifically, we here propose to target the DLPFC, as it is involved in a number of higher-level behaviors and cognitive processes (36, 37) as well as the AG, that occupies a key neuroanatomical position within the parietal structures of the default mode network, a specific network that has been shown to correlate with the level of responsiveness in DoC patients (38, 39).

## Study objectives and hypotheses

Although the choice of stimulation site is becoming an increasingly important issue in the neurostimulation community, no clinical RCT has been performed to determine which stimulation site might be associated with the best clinical outcomes after severe brain injury. Hence, this is the study’s main objective. In a second phase, we will provide a patient-tailored individualized therapy approach through a personalized medicine design. We aim to (1) improve the functional recovery of patients with DoC using either frontal or parietal rTMS, (2) investigate the neurophysiological effects of rTMS interventions in these two distinct brain areas using resting state electroencephalography (EEG) and TMS-EEG, (3) determine the phenotype of clinical responders (i.e., that is, any patient who displayed new sign(s) of consciousness following stimulation that was never displayed during the screening phase nor at baseline), and (4) assess the long-term efficacy of the rTMS interventions in terms of functional outcomes through follow-up assessments.

Our primary hypothesis for the RCT crossover is that a significant portion of our patient sample will show increased responsiveness (i.e., overall CRS-R scores and signs of consciousness) following a single session of DLPFC or AG rTMS (responders). We also expect some patients to show stronger EEG connectivity at the whole brain level, especially in frontoparietal regions (20) compared to sham stimulation.

As for the second phase of the study, as a primary outcome, we hypothesize that patients stimulated over the left DLPFC or the left AG for 20 consecutive sessions will show higher levels of behavioral improvement compared to patients in the sham stimulation group.

Moreover, as secondary hypotheses, we expect that these changes in responsiveness will be associated with modifications in brain complexity and functional connectivity. We postulate that the EEG resting state metrics (e.g., spectral power metrics, connectivity) in the frequencies of interest (i.e., delta, theta and alpha bands) and the TMS-EEG derived measures of brain responses (e.g., PCI) will be modulated by the rTMS intervention and associated with behavioral responses to therapy.

As exploratory hypotheses, we also expect that MCS patients will be more likely to respond to the treatment than patients in UWS/VS. We also expect patients who received the active treatment to obtain better outcome at 3 and 6 months following the end of the intervention period compared to the sham group. No adverse event is expected in any of the three study arms.

## Methods

### Design

This multicenter study consists of two parts: a within-subject, four-arm crossover double-blind RCT and a three-arm parallel double-blind personalized & randomized controlled trial. Both parts will be conducted at the neurological rehabilitation centers William Lennox (Ottignies-Louvain-la-Neuve Belgium), Therapiezentrum Burgau (Burgau, Germany), and Schön Klinik Bad Aibling-Harthausen (Bad Aibling, Germany). A pilot has already been conducted to assess our methods and the protocol as well as our ethical committee have been adapted based on that early testing phase. Therefore, the trial will be preceded by a new pilot phase on a minimum of two patients with the current study design to re-assess feasibility as well as tolerability of our protocol.

### Population and recruitment

Ninety patients with DoC after severe brain injury will be included in the study. Written informed consent will be obtained from patients’ legal surrogates and the patients themselves if they recover functional communication. The study will be conducted in accordance with the Declaration of Helsinki in its latest form. The study protocol was approved by the University Hospital of Liege Ethics Committee under the reference number 2019/277 (BE021921888) and the Ethics Committee of the Medical Faculty at Ludwig-Maximilians-University Munich under the reference number 20–0873 (Therapiezentrum Burgau and Schön Klinik Bad Aibling-Harthausen) and registered on Clinicaltrials.gov (identifier NCT04401319).

Eligibility will be derived from medical records and clinical visits. Inclusion criteria will be the following: patients with DoC due to acquired brain injury classified according to international guidelines as UWS/*VS* or MCS with at least two repeated behavioral assessments with the CRS-R within 10 days prior to inclusion; ≥ 18 years old; > 28 days post-injury; and stable vital parameters. As for exclusion criteria, they will be the following: no previous neurological deficits prior to the brain lesions; no pregnancy; no contraindication for TMS (e.g., uncontrolled epilepsy, that is, seizure within 4 weeks prior to enrollment, metallic implant in the skull, pacemaker, craniotomy under the stimulated site, peri-ventricular shunting device, sensitive skin); no sedative drugs or drugs thought to interfere with brain stimulation such as Na or Ca channel blockers (e.g., carbamazepine) or NMDA receptor antagonists (e.g., dextromethorphan); no drugs or substances which have strong potential of seizure induction (imipramine, amitriptyline, doxepine, nortriptyline, maprotiline, chlorpromazine, clozapine, foscarnet, ganciclovir, ritonavir, phencyclidine, ketamine, gamma-hydroxybutyrate, alcohol, and theophylline); and no current enrollment in other therapeutic clinical trial for the whole duration of the treatment protocol and follow-up. Patients will still receive all the standard medical and para-medical care from their facilities such as sensory stimulation or physical therapy. Table 1 summarizes all inclusion and exclusion criteria. In the present study, we will not exclude patients with lesions at the stimulation site (i.e., over the DLPFC or the AG) as it will enable us to document if patients with this structural profile show a lower rate of clinical responders compared to patients with healthy brain tissue at the target location.

**Table 1:** Study inclusion and exclusion criteria.

Inclusion criteria	Exclusion criteria
Age ≥ 18 years old	Previous neurological deficits prior to the brain lesions
Acquired cerebral damage of known etiology	Pregnancy
Diagnosed in UWS/*VS* or MCS as defined by at least two CRS-R assessments performed during the screening period Time since injury >28 days	Contra-indication for TMS (e.g., uncontrolled epilepsy, that is, seizure within 4 weeks prior to enrollment, metallic implant in the skull, pacemaker, craniotomy under the stimulated site, implanted brain device)
Informed consent given by the legal surrogate	Sedative drugs or drugs thought to interfere with brain stimulation such as Na or Ca channel blockers or NMDA receptor antagonists Concurrent enrollment in any other therapeutic experimental trial

### Procedure

#### Screening phase & enrollment

The study procedure will start at the earliest on the 28th day post-injury. All patients will be evaluated repeatedly (i.e., at least twice within 10 days prior to inclusion) with the CRS-R to confirm DoC diagnosis before enrollment. Existing CT or MRI-scans will be used to document structural lesions for neuronavigation-based targeting within 28 days before inclusion. Following screening phase, the legal surrogate of each eligible patient will be contacted for oral and written informed consent. After inclusion, every patient will first be enrolled in the crossover RCT protocol and will thus undergo four rTMS sessions. Based on the analysis of the patient’s best behavioral or electrophysiological response to either left DLPFC or left AG stimulation, the personalized protocol (4 weeks) will be started up to a week later.

#### Randomized crossover trial

**Figure 2:**
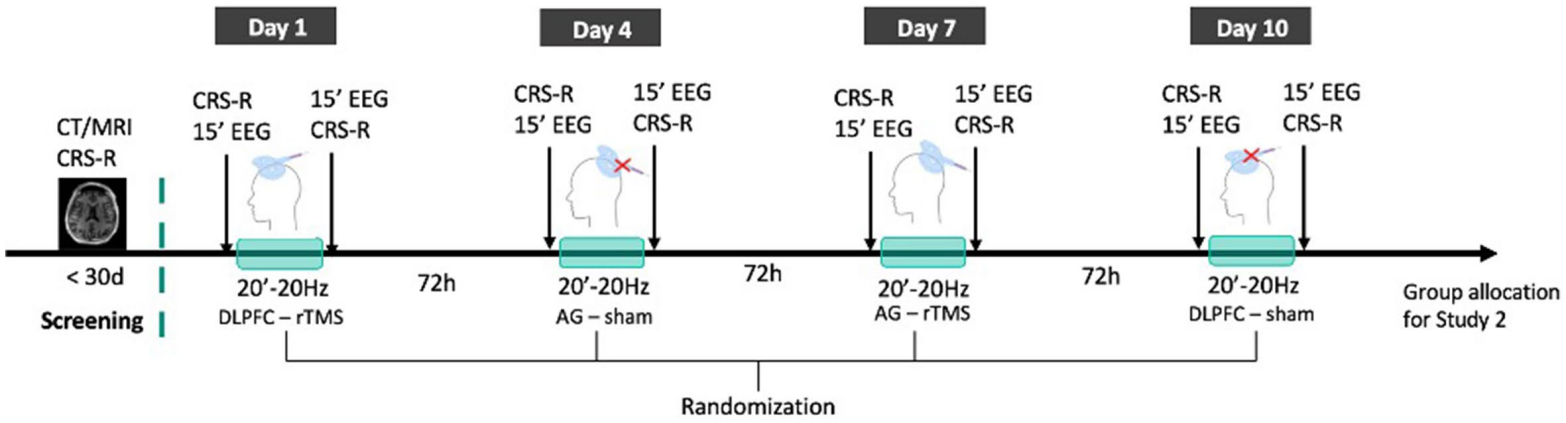
rTMS crossover RCT protocol. Patients’ state of consciousness will be repeatedly assessed with the CRS-R to confirm DoC diagnosis and existing MRI or CT images will be collected. All patients included will then receive 4 sessions (frontal rTMS; parietal rTMS; frontal sham: parietal sham) of 20 min 20 Hz rTMS administered in a double-blind and randomized order within 10 days and separated by a 72 h washout period. Each session will be directly preceded and followed by CRS-R assessments and 15 min resting state EEG recordings. Patients will then be allocated to one of the groups of Study 2.

Within 10 days, all patients will undergo four rTMS sessions that will be administered in a randomized order and separated by a 72 h washout period: (i) one real stimulation over the left DLPFC, (ii) one real stimulation over the left AG, (iii) one sham stimulation over the left DLPFC, and (iv) one sham stimulation over the left AG. In this study, we chose to stimulate the left hemisphere because it tends to be more often targeted in non-invasive brain stimulation trials with DoC patients than the right hemisphere and because it was shown to have more promising results in other top-down electromagnetic-based techniques such as transcranial direct current stimulation (40). Randomization of the sequence of the four stimulation sessions will be stratified for gender, etiology, time post-injury, and diagnosis using computerized random number generator. Standardized behavioral assessments will be performed by experienced clinicians who will be blind to the nature of the sessions. The CRS-R will be performed before and after each stimulation session. Fifteen minutes of resting state high-density EEG will be performed directly before and after the stimulation (i.e., after the behavioral evaluation pre-stimulation and before the behavioral evaluation post-stimulation). Together with the EEG, electrooculogram (EOG) and electrocardiogram (ECG) will be recorded. See Figure 2 for the randomized crossover trial protocol.

#### Personalized parallel trial

**Figure 3:**
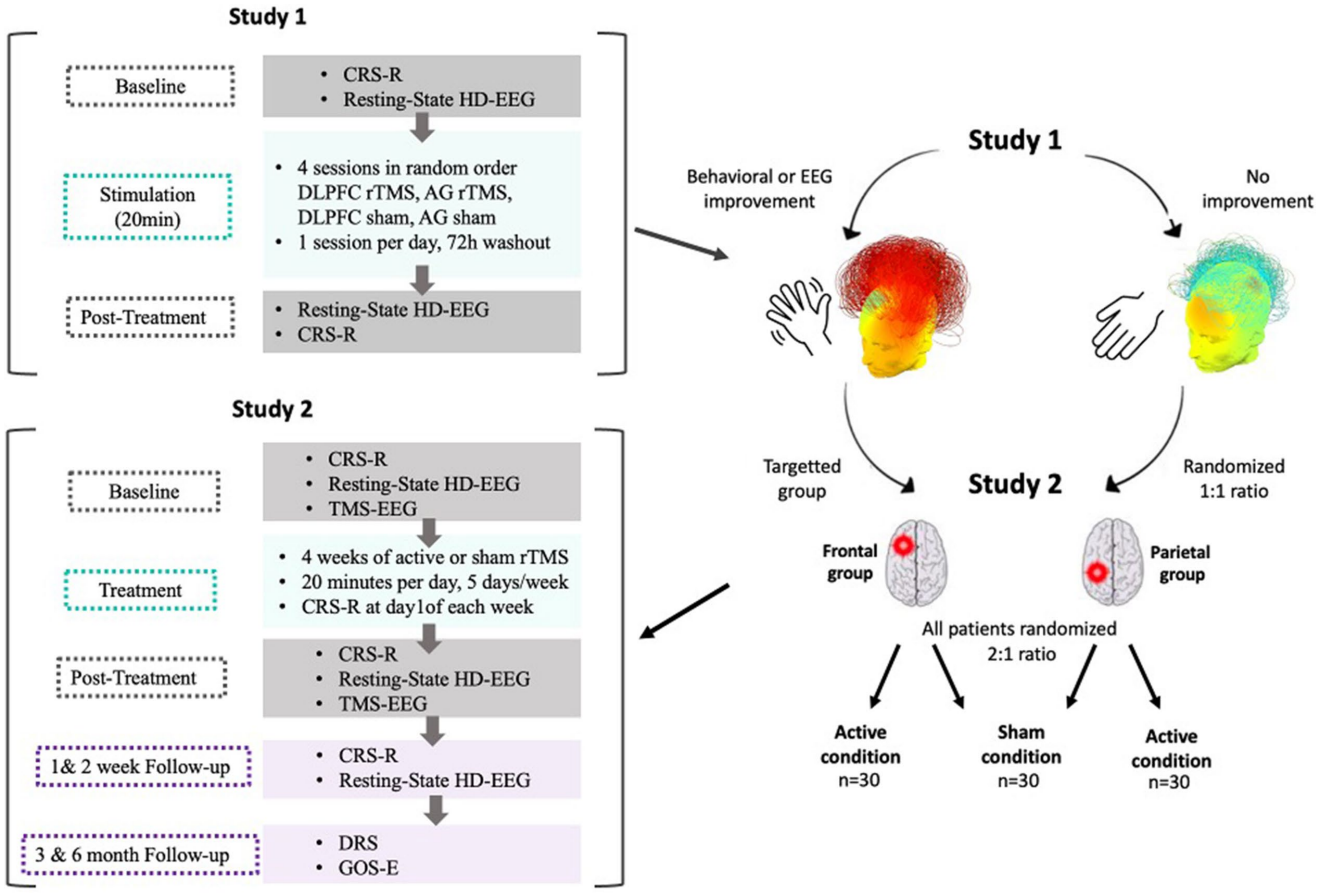
Overall protocol overview. After Study 1, patients who showed behavioral or EEG improvements following stimulation (either frontal or parietal) will be attributed to the corresponding group in Study 2. Patients who showed no improvement following either stimulation types will be randomized between the frontal and the parietal stimulation group in a 1:1 ratio. All patients will then be randomized between active and sham conditions in a 2:1 ratio. In Study 2, patients will be randomly assigned to either the active or the sham condition and will receive a longitudinal treatment protocol. The treatment phase will consist of stimulation sessions applied 20 min per day, 5 days a week during 4 weeks for a total of 20 stimulations sessions. Baseline (first day of stimulation, before the session) and post-treatment (last day of stimulation, after the session) assessments will include the CRS-R, 15 min resting state EEG and TMS-EEG to compute PCI. Follow-up measures (CRS-R and resting state EEG) will be collected at +1 and + 2 weeks following the end of the treatment phase. At 3 and 6 months following the end of the treatment phase, functional outcomes will be collected.

This trial will include three arms (i.e., DLPFC rTMS, AG rTMS and the sham-controlled condition). After a 1 week washout period following the randomized crossover study, the personalized parallel trial will be conducted. Based on their behavioral (primary) or electrophysiological (secondary) responses to either the left DLPFC or left AG treatment in the crossover RCT, patients will be assigned to one of two groups (i.e., DLPFC group if the patient was a responder to the DLPFC stimulation; AG group if the patient was a responder to the AG stimulation). If no behavioral response nor EEG change from the RCT could be obtained regarding the best stimulation hotspot or if the patient is a responder to both sites, the patient will be randomized into one of the two stimulation hotspots in a 1:1 ratio. Then, all patients will be randomized between the experimental condition and the sham condition following a 2:1 ratio by a randomized order generator. Only the investigator in charge of the randomization will be aware of the patients’ group allocation. The assigned intervention (i.e., active stimulation versus sham stimulation) will be concealed from the patient, the family, the care providers and all investigators involved in the patient’s assessment for the whole duration of the treatment phase. The evaluator will stay blind from the sequence as well as from the stimulation group during treatment and follow-up. Moreover, analyses will be conducted in a triple blind fashion (see *rTMS device* point for more information about blinding methods). All patients included in the trial will undergo 4 weeks (i.e., 20 working days) of stimulation. Behavioral effects will be assessed with the CRS-R at baseline and once a week during the 4 weeks stimulation protocol. Fifteen minutes of high-density EEG resting state will be performed right before and right after the first session as well as before and after the last session. Eventually, TMS-EEG acquisitions will be performed the first and last day of the 4 weeks protocol. As we will assess the effects following a single session (first stimulation session) and after 4 weeks of rTMS, we will be able to compare the effect of a single versus repeated sessions of stimulation. Figure 3 depicts an overview of the two studies.

#### Assessment of adverse events & follow-up phase

Throughout both trials, all observed adverse events will be reported, described, and graded on a scale from 1 to 5 (1. mild, 2. moderate, 3. severe, 4. life-threatening, and 5. death referred to as severe adverse event). We will evaluate the proportion of patients who had adverse events and confront them with available adverse effect rates reported in the literature. Following the end of the 4 weeks treatment period, all patients will undergo behavioral (i.e., CRS-R) and neurophysiological (i.e., resting-state EEG) assessments 1 and 2 weeks after treatment to monitor immediate aftereffects. At 3 and 6 months timepoints following the end of the treatment, patient’s functional outcome will be collected. These evaluations will be carried out by means of structured phone interviews with the patient’s relatives/caregivers using the Disability Rating Scale (DRS) (41) and the Glasgow Outcome Scale-Extended (GOS-E) (42).

### Instruments

#### rTMS device

Each stimulation session from the crossover and the longitudinal studies will last 20 min with a frequency of 20 Hz (train duration: 4 s; inter-train interval: 26 s; 3,200 pulses at 120% of the resting motor threshold – RMT, or sham stimulation), adapted from the parameters reported by Legostaeva and colleagues (24) who performed rTMS over the AG in patients with DoC. The RMT (i.e., the minimum stimulus intensity that generated a motor evoked potential response of at least 50 μV at rest for 5 out of 10 trials) will be calculated using single pulses on the corresponding hemisphere of the patient’s dominant hand and reported by a visually detectable twitch in the abductor pollicis brevis muscle. The RMT will be determined at the beginning of each week of treatment with a dedicated round coil, to account for potential changes in the RMT. If a patient presents a high degree of spasticity, or is plegic in the dominant hand, or for any other reason resulting in abnormal corticospinal excitability [which has been described in DoC patients (43)], the other hemisphere will be used for RMT assessment as the RMT calculation is not thought to significantly differ from one hemisphere to the other (44, 45). If the RMT assessment is not conclusive at all, we will rely on RMT data in DoC patients arising from Lapitskaya et al. (43). The associated mean value reported in this article as the mean percentage (%) of maximal stimulator output will be used (i.e., 60%). For the rTMS sessions at both sites, a biphasic stimulator with a capacity up to 100 Hz stimulation will be used (DEYMED diagnostic s.r.o., Hronov, Czech Republic). Stimulations will be delivered through a figure-eight coil with active liquid-cooling (at the Belgian recruiting site) or air-cooling (at the German recruiting sites). Depending on the experimental condition, different coils are used: active stimulation will be delivered via an active rTMS coil, whereas sham stimulation will be delivered via a dedicated sham coil, using the very same parameters. This coil uses a particular shielding mechanism so that no vertical magnetic field is induced. In addition to blocking of magnetic field, the construction of the sham coil makes it suitable for double-blind protocols: since the coil looks the same as the active coil, neither the experimenter nor the patient can see a difference between the coils. The same applies for the acoustic and sensory effect, as there is no difference in the click sound nor in the somatosensory effects. For blinding purposes, a number will be attributed to each coil by the investigator in charge of the randomization. Only the investigator who generated the number assignment will be aware of the allocation and will then disclose the assigned coil number to the investigator in charge of the stimulation the day of the first session.

Structural MRI scans will be used in the crossover RCT to accurately localize the DLPFC and AG (46), using a neuronavigation system that will be connected to the rTMS device (Polaris Vega ST, NDI, Ontario, Canada). In patients without a structural MRI scan, neuronavigation will be performed using a CT scan. In case neuronavigation is not available (e.g., if CT or MRI images could not be obtained), we will target the stimulation area using the 10–20 EEG system electrode positions. The left DLPFC (BA9) can be reached by placing the coil over F3 while the AG has been reported to correspond to BA39, which is under electrode position P3 (47). The cortical structures normally lie within 2 cm of the positions, resulting in 90% accuracy of hotspot detection (48).

Patients are expected to be awake (eyes open) during the rTMS stimulation sessions. If a patient falls asleep, the stimulations will be paused, and the patient will be aroused by auditory or tactile stimulation first. If the patient is still not opening the eyes, the arousal facilitation protocol will be applied according to the CRS-R guidelines (49). The stimulation will resume when the patient opens the eyes again and stimulation time will be adapted accordingly. The patient’s state of arousal will be reported in the Case Report Form for each stimulation session.

#### Behavioral assessments

The CRS-R consists of 23 items arranged hierarchically and divided into six subscales (auditory, visual, motor, oromotor/verbal, communication, and arousal) that test for arousal and awareness in DoC patients (49). The score is based on the presence or absence of behavior in response to stimuli. The total quantitative score is calculated in addition to the best response observed in each subscale. The diagnosis is based on the nature of the best responses observed overall. To overcome the limitation of overlapping total scores for two different diagnoses, the CRS-R index score will be used as well for statistical purposes (50). The validated version for German and French speaking patients will be used accordingly in the study sites (51, 52). The Disability Rating Scale (DRS) is a tool for the quantitative assessment of the severity of brain injuries. It consists of four categories: arousal and awareness, cognitive abilities, physical and psychosocial independence (41). The Glasgow Outcome Scale – Extended (GOS-E) is an interview tool rating the severity of the cognitive, physical, and psychosocial consequences after severe brain injury in the form of an interview with the primary caregiver. It rates the functionality of the patient from death to complete remission (42).

#### EEG

High-density resting state EEG will be recorded using a BrainVision device (BrainAmp, BrainProducts, GmbH, Gilching, Germany). EEG signals will be measured in microvolts, sampled at 500 Hz and referenced to the vertex (Cz) using 64-channels TMS-compatible EEG nets. During the 15 min of recordings in Study 1 and 2, patients will be kept awake (e.g., eyes open) by the experimenter. EEG signals are sensitive to movements and DoC patients are often unable to comply with the instruction to stay still during the recordings. Therefore, in case of agitation or heavy artifacts, recording times will be adapted to obtain enough data to perform the analyses. The resting state data will be used to obtain spectral power and brain connectivity using the graph theory, which have proven to correlate with behavioral recovery of patients with DoC (35).

#### TMS-EEG

The simultaneous use of TMS with EEG implies the perturbation of the brain with a magnetic pulse while recording brain activity electrophysiologically in response to the stimulation. TMS-EEG has become a promising tool in assessing different brain states and functionality (e.g., neural plasticity) over the past two decades (46, 53). The PCI is a mathematical index that expresses the complexity of the brain response to the magnetic perturbation and can successfully discriminate between different brain states (31, 54, 55). In the present protocol, the PCI will be used as a secondary outcome to determine the neurophysiological effects of the 4 weeks rTMS intervention. For TMS-EEG measurements, the coil will be positioned over the premotor area and the precuneus using neuronavigation based on the individual’s T1-weighted MRI or CT images. The stimulation will be individualized depending on the brain responses (first peak-to-peak around 10 μV, and 0.4–0.5 Hz frequency). The jittering of the perturbation (2–2.3 s) should avoid patients building up habituation effects regarding the repetitive stimulation. Once a spot has been found to give appropriate responses as displayed by the general user interface of the machine, a total of 300 pulses will be applied per area (i.e., premotor area and precuneus), which results in a protocol duration of approximately 10 min per area. Noise-masking will be applied via in-ear headphones to avoid auditory late cognitive potentials due to the magnetic stimulation. Moreover, if somatosensory artifacts were to be detected, a thin foil would be placed between the coil and the scalp to reduce skin movement induced by the field.

### Power calculation

There are currently no RCTs available in the literature simultaneously testing the effects of DLPFC and AG rTMS in patients with DoC. As no clear-cut information could be drawn from the literature, a dedicated power analysis was done using G*Power software (56). Assuming a medium effect size of *f* = 0.5 at an alpha error level of 0.05 and a power of 0.8 with ANOVA or multiple regression analysis, 74 patients need to be recruited to detect meaningful differences in the primary outcome (CRS-R) between the real versus sham stimulation groups. Considering a 20% dropout ratio, the number of patients to be recruited adds up to 90.

### Electronic data collection and management

All data collected during this study will be processed and anonymized by an identification number which code will only be known by the researchers involved in the study and will therefore be handled confidentially. Electronic data will be protected by firewall. The researcher in charge will keep the personal data in a file dedicated to the study. All data will be stored and shared between institutions onto Research Space RSpace^©^ – an online secured server providing database security and protection against malicious use. These case report forms (CRF) will be filled out in print and safely stored in lockers inside the clinic and only accessible to the research staff. Patient data will be pseudonymized in all CRF files. These data will be the subject of presentation and scientific publications, in which the identity of the participating patients will be anonymized.

### Data analysis

#### Primary outcomes

For both crossover and longitudinal trials, primary analyses will focus on the detection of behavioral changes (i.e., enhanced behavioral total scores and/or changes in the level of consciousness as defined by the CRS-R) at the group level, comparing the sham interventions to the active interventions; and at the individual level, comparing post treatment to pre-treatment data. Along the same lines, analyses will also offer a comparison of the effects of frontal and parietal active rTMS on patients’ behavioral scores following one session of stimulation for the crossover RCT, and 4 weeks of stimulation for the longitudinal trial. Behavioral CRS-R total scores and subsequent index scores (50) will be defined as our primary outcome. Group treatment effects will be assessed with calculation of the difference between each group post-treatment and pre-treatment score means. Furthermore, we will identify clinical responders to (1) a single session of rTMS in the crossover trial and (2) the 4 weeks treatment protocol of the longitudinal trial as patients who will display new sign(s) of consciousness following treatment that was not present at baseline nor during the screening phase. In that context, further subgroup analyses will also be conducted along age, etiology, time since injury and diagnosis at inclusion.

#### Secondary outcomes

In the RCT, the change in EEG metrics between post and pre stimulation of each session will be estimated and will stand as our secondary outcome. More specifically, analyses will focus on changes in whole brain connectivity markers as well as on power spectrum for each frequency band and brain response complexity. The alpha-band participation coefficient will be used to determine the response of a patient to the stimulation in the crossover stimulation protocol by means of pre and post stimulation differences. The same metrics will be computed and compared for the longitudinal trial before and after the 4 weeks treatment period. Additionally, for the latter study, TMS-EEG derived PCI will be computed and compared using the same method. TMS-EEG data will be analyzed with EEGLAB,^1^ FieldTrip,^2^ Brainstorm,^3^ MNE-Python^4^ and in-house MATLAB (SSP BioMedical Data Analysis Package; SiSyPhus Project; Version 2.5e) and Python scripts. The resting state data will be analyzed with a dedicated analysis pipeline (35). Continuous EEG resting state data will be filtered between 0.5 and 45 Hz and segmented into 10 s epochs. Then, data will be thresholded to remove clear-cut artifacts. EOG and ECG will be used to inform the removal of artifactual data epochs. An independent component analysis (ICA) will be used to remove remaining artefactual components from the EEG signal. Data will be used to compute spectral connectivity in the frontal and parietal areas in the delta, theta and alpha frequencies and expressed in graph theorem-based metrics (e.g., participation coefficient in the alpha band). Further, a set of these graph-theoretic parameters will be extracted from the network analyses and used to train and test a machine-learning model. We will analyze the parameters’ capacity to inform and predict treatment outcome independently (univariate regressions) and combined (multivariate pattern analysis and machine learning).

Data analysis will be carried out using RStudio (57). Analyses will be based on means ± standard deviations (SDs) for normally distributed quantitative variables, and as median and interquartile range (P25 – P75) for the skewed distributed variables. Numeric outcomes (e.g., the number of responders to the 4 weeks rTMS programme) will be summarized using count and proportion (%). Results will be considered significant at the 5% critical level (*p* < 0.05) and will be corrected using Holm correction for multiple comparisons. The Cohen’s d effect size will be calculated from the difference in means and standard deviations between baseline and post-treatment comparing active with sham interventions.

### Dissemination of results

Results of this clinical trial will be published in peer-reviewed open-access journals as original research articles and will be presented at various scientific conferences. The first publication will cover the clinical (CRS-R) and electrophysiological (connectivity markers) results of the RCT. The second publication is planned to report the clinical (CRS-R) and electrophysiological (connectivity markers and PCI) results of the personalized clinical trial and the follow-up period. A third publication is reserved for a detailed description of the machine-learning classifier developed to determine the features of treatment responders.

## Discussion

The current state of experimental science and medicine only offers few adequate therapeutic options for patients with prolonged and chronic DoC and their long-term management is becoming a public health concern (4). Moreover, the absence of clear consensus regarding a patients’ prognosis coupled with the lack of therapeutic opportunities may play a critical role in medical care decisions having an undeniable impact on patient’s survival. Because of these issues, it is crucial that more resources be put in place to further verify the potential effect of new therapies in robust settings and define who they might benefit to the most. In that context, some patients with DoC after severe brain injury can be successfully treated with non-invasive therapeutic interventions (7), among which rTMS seems to be the most effective option (25). However, there is currently a debate on whether recovery is mostly supported by the frontal or the posterior networks and structures (26, 58). While there seems to be evidence for the efficacy of targeting both regions with rTMS in promoting behavioral and/or electrophysiological recovery in patients with DoC (13, 17, 24, 59, 60), this protocol describes, to the authors’ knowledge, the first study investigating a direct comparison of the frontal versus parietal theories of stimulation hotspots (26). This clinical trial could help to understand which stimulation hotspot for non-invasive brain stimulation with rTMS is best suited for a patient. While the best research designs to support treatment efficacy in a given population are indisputably RCT, it becomes more and more evident that the field of therapeutic management of DoC patients is guided towards the direction of a personalized treatment approach instead of systematic randomization (61–63). Indeed, as described earlier, significant positive results are rarely observed in all DoC patients following non-pharmacological interventions. This suggests that not all patients can benefit from all types of interventions, thus supporting the clinical approach which pays particular attention to each patient characteristics and potential positive response to treatment in order to design a treatment plan. Thus, by positioning itself in that direction, this protocol acts as a major step in the pioneering approach of the development of patient-fitted tailored interventions. Although there is already existing evidence regarding certain endotype markers that may allow for response to brain stimulation treatments, the field is still at its debuts and needs massive joint efforts to provide conclusive guidelines for the clinical setting. In that sense, our personalized approach might help to increase the number of responders as compared to previous RCTs in the literature. Consequently, such increased number of responders will allow us to extract and define a possible phenotype regarding the effectiveness of transcranial magnetic brain stimulation.

The overall goal of this personalized trial is to improve the functional recovery at the clinical level. At the electrophysiological level, this study offers the opportunity to test different models of consciousness: anterior stimulation will allow to study the effect on consciousness recovery (32) according to the fronto-parietal mesocircuit model (28, 29) and the global neuronal workspace theory, suggesting that the hotspot of consciousness is located at the front of the brain (33, 34). Posterior stimulation – on the contrary – will allow for the testing of IIT claiming that the posterior part of the brain is the hotspot of consciousness (26, 27). We will use neurophysiological assessments as well as neurobehavioral exams to test the hypothesis that rTMS can modulate the neural network of the severely injured brain to promote the recovery of both consciousness at the clinical level, and functional thalamocortical network integrity at the neurobiological level. As such, this trial will bring direct evidence to challenge the above-mentioned models and will shed new light on the use of frontal and parietal rTMS as a therapeutic candidate to treat DoC.

A potential pitfall of this protocol might be the challenging timeframe of investigation. Indeed, full completion of the procedure of both trials should add-up to a total of 9 weeks. This is a particularly challenging feature since as we mentioned earlier, DoC patients are a very fragile population prone to complications and management issues. Safety precautions will be taken to avoid potential harm to the patients during the study, especially during the rTMS stimulation sessions, and to allow patients to complete the protocol.

This protocol stands as an important milestone in the development of new patient-tailored therapeutic options in the field of DoC. Our findings could usher in a new era of research for a challenging patient population in desperate need of medical solutions.

## Ethics statement

The studies involving human participants were reviewed and approved by University Hospital of Liège Ethics Committee, William Lennox Neurological Hospital Ethics Committee (Belgian identifier B0201941888), Ethics Committee of the medical faculty of the Ludwig-Maximilians-University Munich (identifier 20-0873). The patients/participants will provide their written informed consent to participate in this study.

## Author contributions

MV, MR, MN, AB, and OG were involved in conception and methodology design of the study, ethical and trial registration procedures, and manuscript writing. MV, MR, OG, and AB lead the implementation and the coordination of the trials. PC, LW, NL, and AT participated in trial methodology design and provided technical expertise regarding the clinical trial materials and settings. AT, SL, AB, and OG participated in study conception and helped defining its theoretical framework. All authors contributed to the article and approved the submitted version.

## Funding

This work was supported by the ZNS Hannelore-Kohl Stiftung and the Federal Ministry of Education and Research (BMBF), the Belgian National Funds for Scientific Research (FRS-FNRS), FNRS project No PDR/BEJ T.0134.21, the University of Liège Conseil Sectoriel de la Recherche, the European Union’s Horizon 2020 Framework Programme for Research and Innovation under the Specific Grant Agreement No. 945539 (Human Brain Project SGA3), the ERA-Net FLAG-ERA JTC2021 project ModelDXConsciousness (Human Brain Project Partnering Project), the European Space Agency (ESA) and the Belgian Federal Science Policy Office (BELSPO) in the framework of the PRODEX Programme, the GIGA Doctoral School for Health Science, the BIAL Foundation, the Mind Science Foundation, the fund Generet of the King Baudouin Foundation, the Mind Care International foundation and AstraZeneca Foundation. SL is FNRS Research Director, OG and AT are FNRS Research Associates, NL is FNRS Post Doctorate Fellow, and MV and PC are FNRS Research Fellows.

## Conflict of interest

The authors declare that the research was conducted in the absence of any commercial or financial relationships that could be construed as a potential conflict of interest.

## Publisher’s note

All claims expressed in this article are solely those of the authors and do not necessarily represent those of their affiliated organizations, or those of the publisher, the editors and the reviewers. Any product that may be evaluated in this article, or claim that may be made by its manufacturer, is not guaranteed or endorsed by the publisher.

## Footnotes

^1^https://sccn.ucsd.edu/eeglab/

^2^http://www.fieldtriptoolbox.org/

^3^https://neuroimage.usc.edu/neuro/BrainStorm

^4^https://mne-tools.github.io
